# Combined warming index energy system analysis framework for methane leakage rate and carbon capture rate uncertainty^☆^

**DOI:** 10.1016/j.mex.2025.103526

**Published:** 2025-07-23

**Authors:** Daniel Davids, Neil Grant, Shivika Mittal, Adam Hawkes, Gbemi Oluleye

**Affiliations:** a Department of Chemical Engineering, Imperial College London, London, SW7 2AZ, United Kingdom; b Grantham Institute for Climate Change and the Environment, Imperial College London, London, SW7 2AZ, United Kingdom; c CICERO Centre for International Climate Research, Oslo, 0349, Norway

**Keywords:** Hydrogen, Blue hydrogen, Hydrogen economy, Methane leakage rate, Carbon capture rate, Combined warming index, Energy systems modelling, Integrated Assessment Modelling, Decarbonisation, Net-zero

## Abstract

Fossil fuels dominate the production of hydrogen and will continue to contribute in a decarbonised future. Blue hydrogen production from natural gas with carbon capture and storage technology applied is seen as the major route for natural gas in a future Hydrogen Economy. Methane leakage rate in natural gas supply chains and carbon capture rate are two critical parameters for the success of blue hydrogen. Despite this, the linked effect of the variables are difficult to identify, especially in terms of their impacts on decarbonisation metrics within the energy system. We formulate a new Combined Warming Index (CWI) measure and develop a framework for analysing the influence of methane leakage rate and carbon capture rate on blue hydrogen viability and other relevant energy system characteristics.

Framework outline:

• Investigate energy system scenarios within a range of methane leakage rates and carbon capture rates (varying Combined Warming Indices [CWI]) on blue hydrogen.

• Analyse important energy system dynamic parameter indicators versus the Combined Warming Index (CWI).

• Resultant energy system trends for methane leakage rates and carbon capture rates analysed against Combined Warming Index (CWI) establish unique property envelopes that reveal the state of the energy system at conditions and periods of interest.

## Graphical abstract

**Figure:**
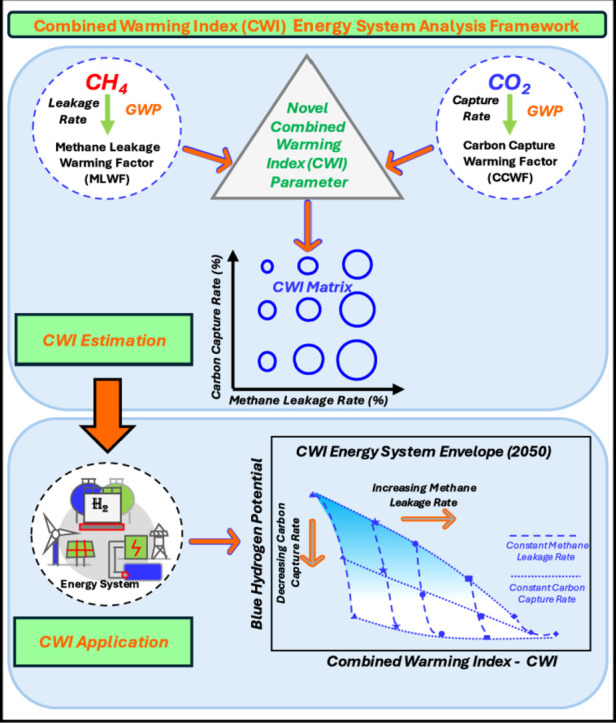

## Specifications table

**Table:** 

**Subject area**	Energy
**More specific subject area**	Energy System Modelling and Integrated Assessment Modelling Analysis
**Name of your method**	Combined Warming Index (CWI) Energy System Analysis Framework
**Name and reference of original method**	Not Applicable
**Resource availability**	Not Applicable

## Background

Greenhouse gases, including methane (CH_4_) and carbon dioxide (CO_2_), are some of the main instigators of global warming [1]. Blue hydrogen from natural gas is anticipated to play a key role in the quest to meet global decarbonisation and net-zero goals in line with the Paris Agreement [2], further strenghtened in 2021 [3] which seeks to constrain global temperature increase to sustanably below 2 °C and around 1.5 °C.

Natural gas is primarily composed of methane, a potent greenhouse gas with warming effects and a Global Warming Potential (GWP) of around 25 times more than carbon dioxide which gives it the capability to have more negative influence on the climate. The oil and gas industry accounts for about a quarter of yearly methane emissions [4,5], although there is a wide variance in the assessments of the emissions between 0.003–10 % [6,7] from the subsectors along the oil and gas supply chain, from exploration and production, transportation, storage, to end use. This has potential implicatons for the viability of blue hydrogen and other natural gas or methane based processes within the energy system.

Carbon Capture and Storage (CCS) has been identified as a critical technology for alleviating climate change at least-cost [8,9] and serves as a major tool in decarbonisation scenarios from energy system models and integrated assessment models (IAMs) that are consistent with satisfying targets of the Paris Agreement [10,11].

Additionally, important for our method, CCS may be useful as a form of low-carbon hydrogen (blue hydrogen) production [12,13]. This method of producing hydrogen relies on natural gas by the application of CCS technology to applicable production process such as Steam Methane Reforming (SMR) or Autothermal Reforming (ATR) [14]. Due to the immaturity of CCS as a technology [15], the capability of CCS in processes such as blue hydrogen production mainly determined by how much CO_2_ can be retained is uncertain, with a variable range of 52–97 % established [[16], [17], [18]].

Within the wider integrated energy system, the lack of confidence in the parameters of methane leakage rate and carbon capture rate affects blue hydrogen implementation in energy system models and integrated assessment models (IAMs) for decarbonisation studies, hence net-zero emission scenarios [19]. These parameters are usually focused on individually thereby not capturing the complete environmental and system effects for the related processes [20,21].

Furthermore, the complexities of integrated energy systems further make it challenging to analyse the effect of the uncertainty and variability of methane leakage rate from key supply chains such as oil and natural gas production and subsequent end use sectors or industries, and carbon capture rate for abating environmentally harmful processes and sectors on energy system dynamics and measures across structural segments.

## Method details

Our methodology presents the creation, evaluation and associated protocol for the Combined Warming Index (CWI) parameter.

### Combined warming index (CWI) formulation

The intensity of methane emissions released along the production route from natural gas extraction to utilisation (methane leakage rate) in addition to the capability of carbon capture and storage technology applied to processes (carbon capture rate), in our focus, blue hydrogen generation process from natural gas are uncertain and variable, and are important constraints on this form of low-carbon hydrogen production performance. The impacts of these within the details of whole system energy system models are difficult to establish and analyze.

We relate Global Warming Potential (GWP) [1,22], methane leakage rate from oil and gas processing fugitive and non-fugitive emissions [23,24], and carbon capture rate [16,25] through new measures and equations. The Combined Warming Index (CWI) is evaluated as a two-component indicator. A Methane Leakage Warming Factor (MLWF) and Carbon Capture Warming Factor (CCWF).

The Methane Leakage Warming Factor (MLWF) estimated as:

(1) $$\begin{array}{c} \text{Methane}\;\;\text{Leakage}\;\;\text{Warming}\;\;\text{Factor}\;\left(\text{MLWF}\right)\;{\left(\text{MLWF}\right)}_{|100}=\left(CH_{4}\;\mathrm{Leak}\;\;\mathrm{age}\;\mathrm{Rate}*\text{GW}P_{CH_{4|100}}\right) \end{array}$$

The Carbon Capture Warming Factor (CCWF) evaluated as:

(2) $$\begin{array}{c} \text{Carbon}\;\text{Capture}\;\text{Warming}\;\text{Factor}\;\left(\text{CCWF}\right)\;{\left(\text{CCWF}\right)}_{|100}=\left(\left(1-CO_{2}\;\mathrm{Capt}\mathrm{ure}\;\mathrm{Rate}\right)*\text{GW}P_{CO_{2|100}}\right) \end{array}$$

Our Combined Warming Index (CWI) is expressed as:

(3) $$\begin{array}{c} \text{Combined}\;\text{Warming}\;\text{Index}\;\left(\text{CWI}\right)\;{\left(\text{CWI}\right)}_{|100}={\left(\mathrm{MLWF}\right)}_{|100}+\;{\left(\mathrm{CCWF}\right)}_{|100}\\ =\left(CH_{4}\;\mathrm{Leak}\mathrm{age}\;\mathrm{Rate}*\text{GW}P_{CH_{4|100}}\right)+\left(\left(1-CO_{2}\;\mathrm{Capt}\mathrm{ure}\;\mathrm{Rate}\right)*\text{GW}P_{CO_{2|100}}\right) \end{array}$$

whereGWPistheGlobalWarmingPotential

|100denotes100−yeartimeframe

This parameter sums the effects of methane leakage rate and carbon dioxide capture rate and creates a method to combine the effect of the associated greenhouse gases. We use this metric within a framework for analysing energy system model decarbonisation scenario outputs to reveal the interplay of methane leakage rate and carbon dioxide capture rate for blue hydrogen on energy system variables. It is also possible to apply this methodology to other processes that are based on the natural gas supply chain and CCS technology.

### Combined warming index (CWI) energy system analysis framework

Our procedure for the application of the Combined Warming Index (CWI) consists of two stages, the first estimating a range of Combined Warming Index (CWI) values within brackets of methane leakage rate and carbon capture rate, the second, involving the application to energy system analysis.

### Combined warming index (CWI) estimation

**Fig. 1:**
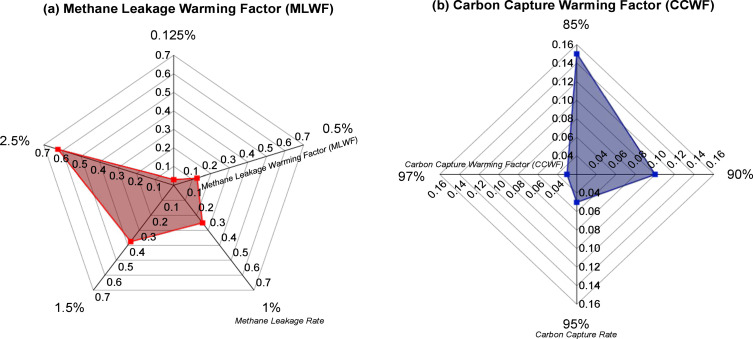
(a) Methane Leakage Warming Factor (MLWF) (b) Carbon Capture Warming Factor (CCWF).

The Methane Leakage Warming Factor (MLWF) is firstly evaluated for a range of methane emission rates utilising the global warming potential (GWP) for methane, Fig. 1(a). Our methane leakage rate accounts for the total supply chain emission of methane involved in the blue hydrogen production process and is of sufficient range to account for natural seepages [26,27] that could be associated with hydrocarbon exploration and production. Following this, Carbon Capture Warming Factor (CCWF) is computed for carbon capture rate values within the scope of the analysis, Fig. 1(b).

**Fig. 2:**
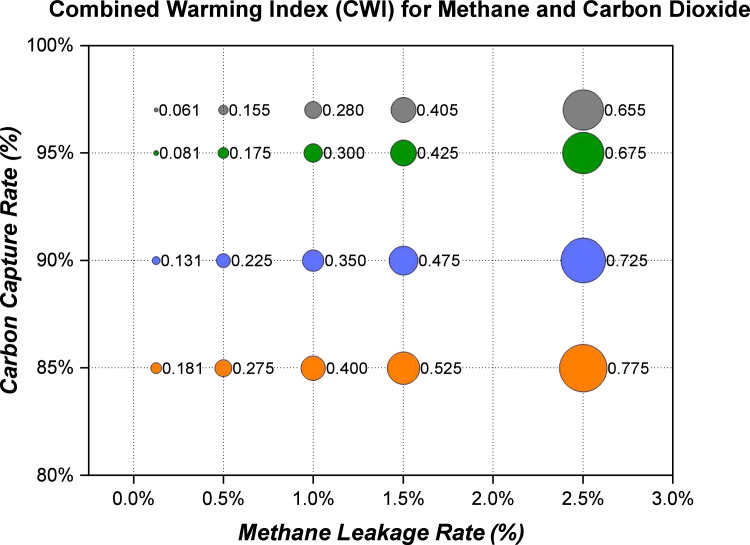
Bubble Plot of Example Combined Warming Index (CWI) Estimation. CH_4_ GWP=25 and CO_2_ GWP=1.

The Methane Leakage Warming Factor (MLWF) and the Carbon Capture Warming Factor (CCWF) are weighted based on the GWP [28] of methane and carbon dioxide respectively and their proportions released from the relevant stages of the blue hydrogen supply chain.^1^ This helps to reveal the greenhouse gas impact of each element that forms the Combined Warming Index (CWI). The Methane Leakage Warming Factor (MLWF) and Carbon Capture Warming Factor (CCWF) are aggregated to provide a matrix of Combined Warming Index (CWI) values across the limits of methane leakage rates and carbon capture rates, Fig. 2.

### Combined warming index (CWI) application

Energy system scenarios are run according to the matrix of methane leakage rate and carbon capture rate combinations across the ranges, hence Combined Warming Index (CWI) values. Principal model results of investigation that give a snapshot in time are plotted against the Combined Warming Index (CWI) values for the cases. The Combined Warming Index (CWI) Energy System Analysis can be performed at several levels; Constant Methane Leakage Rate Trends, Constant Capture Rate Trends and Combined Warming Index (CWI) Energy System Envelopes using energy system parameters at periods of interest. For evaluation at constant methane leakage rate trends, the trends of constant methane leakage rates are studied within the carbon capture range. This helps to highlight the sensitivity of the energy system decarbonisation parameters to carbon capture efficiency at a certain level of methane emissions in the natural gas supply chain. Similarly, when approaching the assessment at constant carbon capture rate trends, the trends of constant capture rates are studied within the bracket of methane leakage being considered. This reveals variability with respect to methane leakage rate when carbon capture rate is stable.

**Fig. 3:**
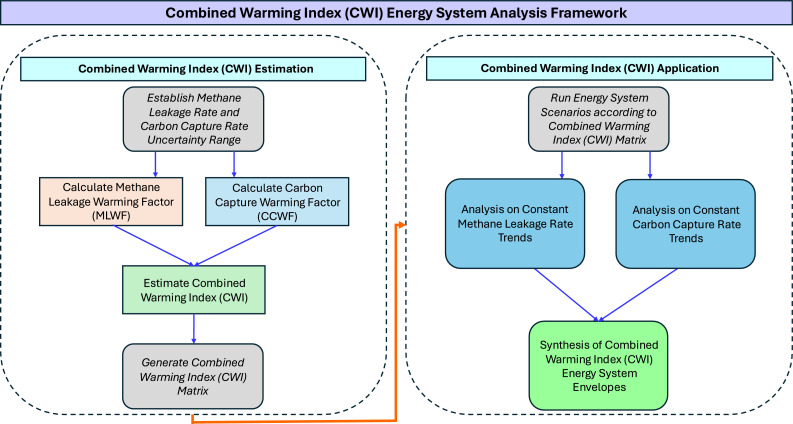
Flowchart of Combined Warming Index (CWI) Energy System Analysis Framework.

The constant Methane Leakage Rate Trends and Constant Capture Rate Trends can be amalgamated to form Combined Warming Index (CWI) Energy System Envelopes. The envelopes can be analysed for various energy system decarbonisation variables and gives an indication of the dynamics of these tests on the changeability of methane leakage rate and carbon capture rate on the integrated energy system. Energy system measurements can be further interpolated within the limits of the Constant Methane Leakage Rate Trends, Constant Capture Rate Trends and Combined Warming Index (CWI) Energy System Envelopes. This can give reliable indicators of the range of variables within the space of methane leakage rate and carbon capture rate without running the full energy system model and post processing results. A summary of the Combined Warming Index (CWI) Energy System Analysis Framework is given in the flowchart in Fig. 3.

The Combined Warming Index (CWI) and its applicable framework has the advantage of bringing into focus the component aspects of methane leakage rate [29] and carbon capture rate [30] which both contribute to the carbon intensity of blue hydrogen.^2^ Scrutiny of these two factors while amalgamated in the Combined Warming Index (CWI) still enables a disaggregation of methane leakage rate and carbon capture rate constituents [31]. This allows the dynamics between methane leakage rate and carbon capture rate within their variable ranges, and their combined effects on blue hydrogen performance and energy system decarbonisation metrics to be evaluated.

## Method validation

**Fig. 4:**
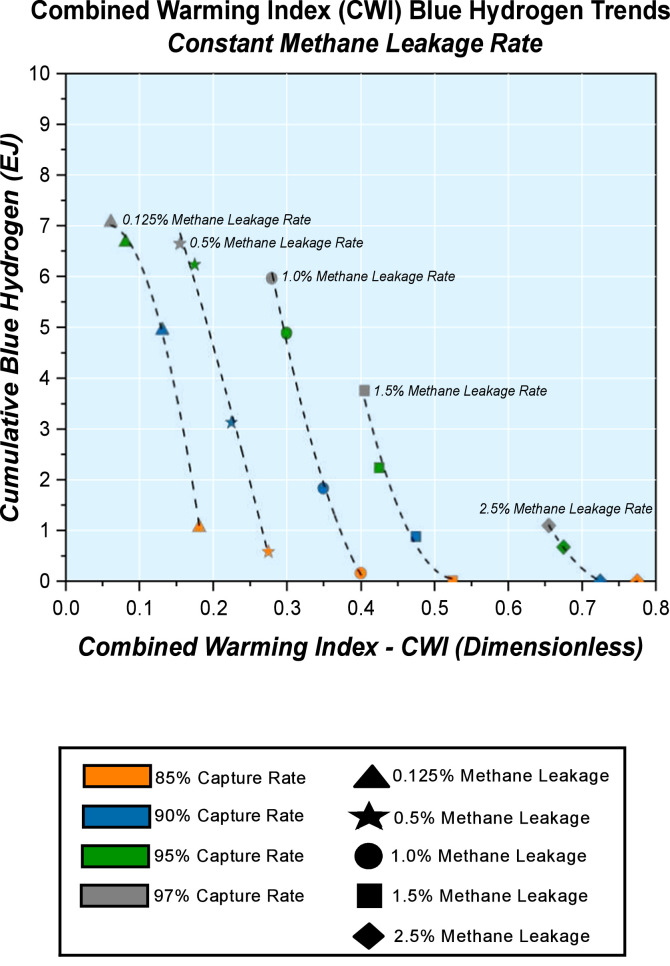
Combined Warming Index (CWI) and Cumulative Blue Hydrogen by 2050 with Constant Methane Leakage Rate Trends.

**Fig. 5:**
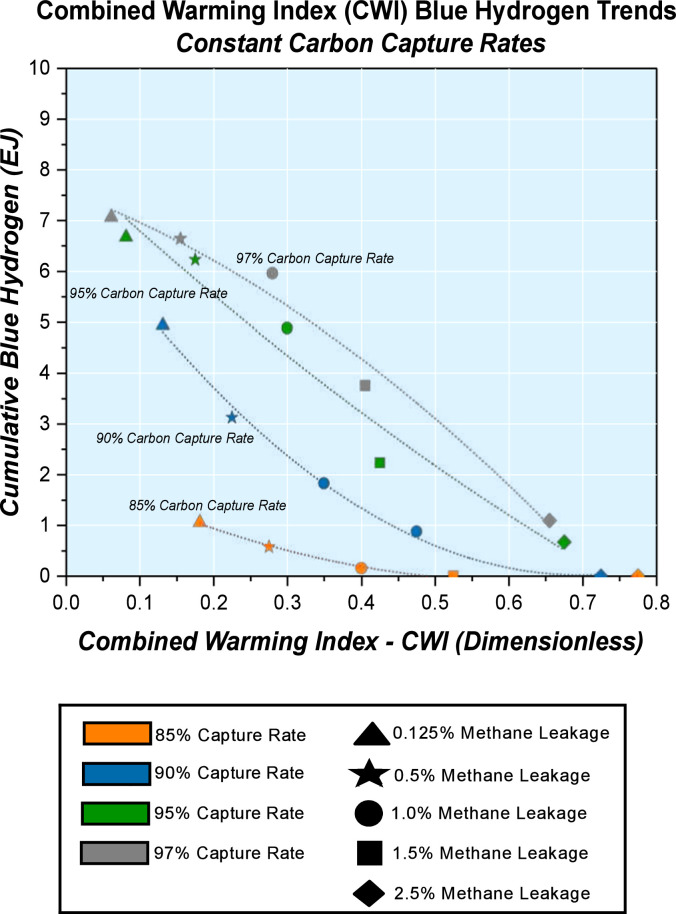
Combined Warming Index (CWI) and Cumulative Blue Hydrogen by 2050 with Constant Carbon Capture Rate Trends.

Data that validates our methodology is presented using outputs from an energy system model of the United Kingdom (UK). This model is based on UK-TIMES MARKAL (UKTM) energy system model [32] associated with the modelling conventions of the International Energy Agency’s Energy Technology Systems Analysis Program (IEA-ETSAP). Scenarios that depict potential progression of the UK energy system and that meet net-zero requirements by 2050 are evaluated within the matrix of methane leakage rates and carbon capture rates from Fig. 2; a total of 20 cases. The impact of upstream methane emissions, represented as the methane leakage rate and differing carbon capture performances of carbon capture and storage solutions linked to related processes for blue hydrogen production is investigated with results subsequently highlighted. In Fig. 4, the Combined Warming Index (CWI) and cumulative blue hydrogen trends by 2050 are shown. These are for analysis at constant methane leakage trends. An alternate assessment basis, with scrutiny based on constant carbon capture rate trends (Fig. 5) is possible.

**Fig. 6:**
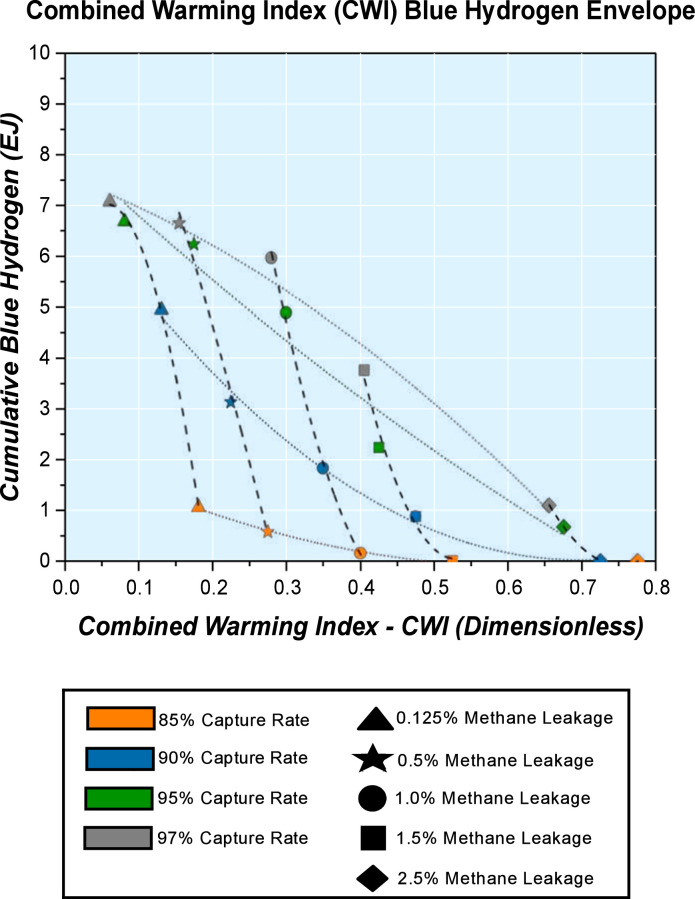
Combined Warming Index (CWI) Blue Hydrogen Envelope indicating the range of deployment possible by 2050 within the constraints of methane leakage rates and carbon capture rates tested for blue hydrogen production within the energy system.

**Fig. 7:**
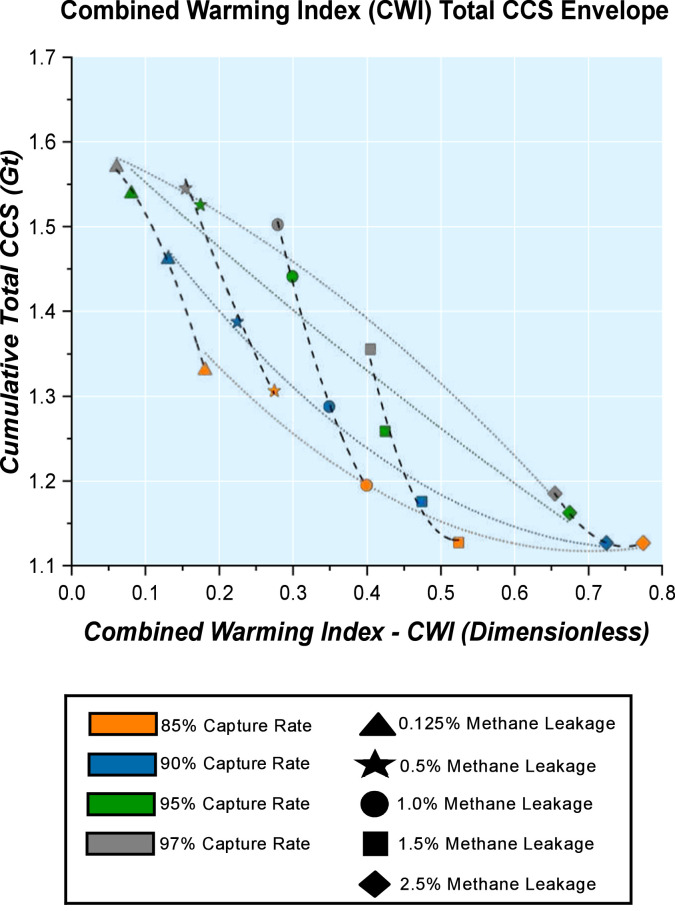
Combined Warming Index (CWI) Total Carbon Capture and Storage (CCS) Envelope showing energy system CSS application by 2050 for methane leakage rates and carbon capture rates studied.

**Fig. 8:**
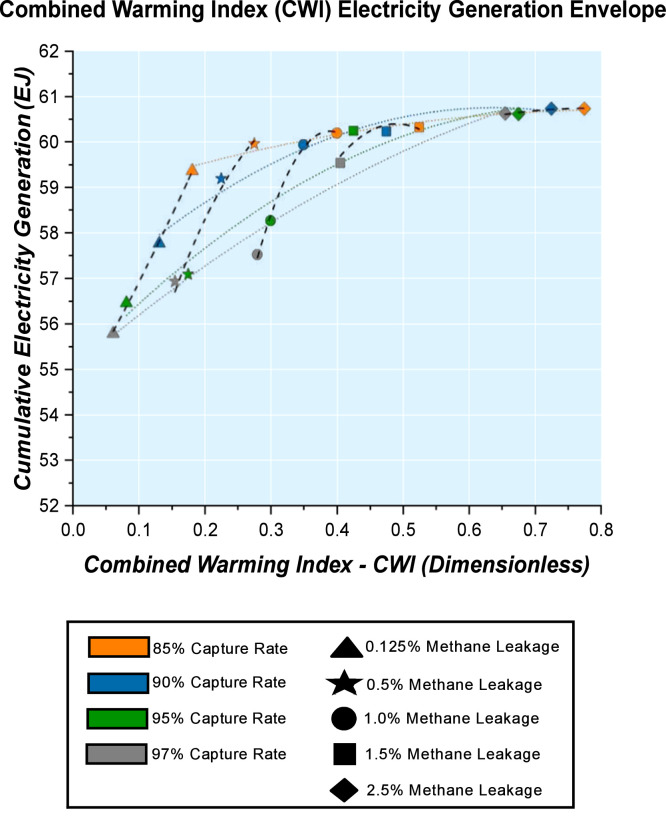
Combined Warming Index (CWI) Electricity Generation Envelope for contibutions of all electricity sources within the energy system by 2050 for methane leakage rate and carbon capture rate sensitivities.

**Fig. 9:**
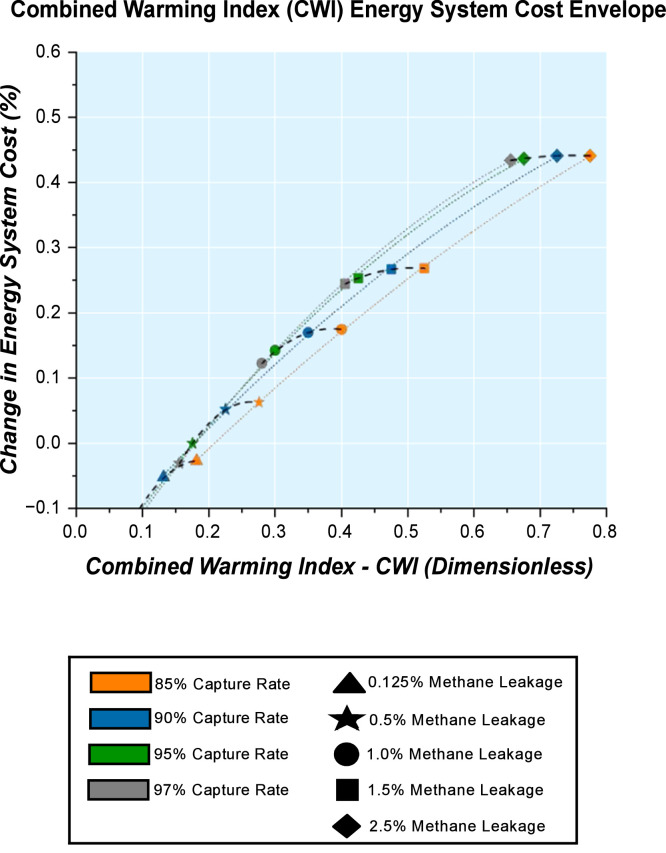
Combined Warming Index (CWI) Energy System Cost Envelope by 2050 for methane leakage rates and carbon capture rates indicated.

Figs. 6–9 reveal some select Combined Warming Index (CWI) Energy System Envelopes. Constant methane leakage trends and constant carbon capture rate trends can be overlapped to give a Combined Warming Index (CWI) Energy System Envelope as in Fig. 6, where a Combined Warming Index (CWI) Blue Hydrogen Envelope is shown.

An amalgamation of constant methane leakage trends and constant carbon capture rate trends for a Combined Warming Index (CWI) Total CCS Envelope is further revealed in Fig. 7.

A Combined Warming Index (CWI) Electricity Generation Envelope with constant methane leakage trends and constant carbon capture rate trends together (Fig. 8) explores the effects of methane leakage rates and carbon capture rates on the dynamics of power generation from all sources and within all sectors the energy system.

The energy system cost is an important metric in energy system analysis, and it reveals the total system cost represented as net present value (NPV) within the energy system. A Combined Warming Index (CWI) Energy System Cost Envelope with constant methane leakage trends and constant carbon capture rate trends together (Fig. 9) highlights the characteristics for the case range appraised. For this, a change in energy system cost with respect to a reference scenario (0.5 % methane leakage rate and 95 % carbon capture rate) is used as a means of comparison across the cases.

Regression analysis can be performed on the constant methane leakage trends and constant carbon capture rate trends to establish relationships at the various levels of analysis. This provides a means of deriving the outputs of Combined Warming Indices (CWI), hence methane leakage rate and carbon capture rate combinations from the regression equations without running intermediary sensitivity cases. Table 1 shows regression parameters for Combined Warming Index (CWI) and cumulative blue hydrogen by 2050 at constant methane leakage rate trends. In Table 2, the regression parameters at constant methane leakage rate trends in presented.

**Table 1:** Combined Warming Index (CWI) and Cumulative Blue Hydrogen by 2050 with Constant Methane Leakage Rate Trends – Regression Parameters.

y = Intercept + B1*x^1 + B2*x^2					
Plot	0.125 % Methane Leakage	0.5 % Methane Leakage	1 % Methane Leakage	1.5 % Methane Leakage	2.5 % Methane Leakage
Intercept	5.79658 ± 0.5977	12.93888 ± 6.80201	41.32318 ± 9.4741	65.02219 ± 28.05345	72.74632 ± 2.73136
B1	43.15201 ± 11.16437	−31.74896 ± 65.82259	−179.34006 ± 56.60186	−246.00931 ± 121.64865	−194.02003 ± 7.66571
B2	−381.75837 ± 45.57175	−48.76242 ± 152.51051	190.84624 ± 83.09535	232.85913 ± 130.66869	129.22736 ± 5.35771
Residual Sum of Squares	0.01804	0.20203	0.05997	0.14831	2.49E-04
R-Square (COD)	0.9992	0.9917	0.99723	0.98167	0.99971
Adj. R-Square	0.99761	0.97511	0.99168	0.94502	0.99914

**Table 2:** Combined Warming Index (CWI) and Cumulative Blue Hydrogen by 2050 with Constant Carbon Capture Rate Trends – Regression Parameters.

y = Intercept + B1*x^1 + B2*x^2				
Plot	85 % Capture Rate	90 % Capture Rate	95 % Capture Rate	97 % Capture Rate
**Intercept**	2.14864 ± 0.12423	7.2868 ± 0.39085	8.1277 ± 1.09682	7.57394 ± 0.62346
**B1**	−7.18228 ± 0.59129	−20.85345 ± 2.13441	−13.66095 ± 7.00422	−5.39254 ± 4.26104
**B2**	5.70819 ± 0.60233	14.96552 ± 2.41563	3.52741 ± 8.91149	−7.0825 ± 5.70334
**Residual Sum of Squares**	0.00513	0.08245	1.12205	0.45959
**R-Square (COD)**	0.99388	0.99454	0.95849	0.98133
**Adj. R-Square**	0.98777	0.98909	0.91699	0.96266

## Limitations

A drastic reduction in greenhouse gas (GHG) emissions, including carbon dioxide (CO_2_) and methane (CH_4_) is pertinent to accomplish the goals of the Paris Agreement which is pursuing global temperature rise constrained lower than 2 °C and additionally targets around 1.5 °C, with increasingly stringent committments [2,3]. The Hydrogen Economy and blue hydrogen have an important roles to play within the decarbonisation landscape. This brings in focus emissions from hydrocarbon supply chains (methane leakage rate) as well as an important effieciency indicator for CCS technology applied to hydrogen prodcution processes from natural gas; the carbon capture rate. Both of these variables are uncertain. This makes it urgent to develop methods and frameworks to test the influence of methane leakage rate and carbon capture rate on the performance of blue hydrogen against the backdrop of the details of the wider integrated energy system.

We develop a novel paramater called Combined Warming Index (CWI) which brings together the effect of methane leakage rate and carbon capture rate. This measure is used within our Combined Warming Index (CWI) Energy System Analysis Framework to analyse the results of energy system sceanrios within a matrix of methane leakage rate and carbon capture rate estimates. The outputs of choice energy system parameters can be analysed at specificed time horizons thus revealing the impacts of methane leakage rate and carbon capture rate, indicated by the Combined Warming Index (CWI) on the energy system. Investigtion may be done at constant methane leakage rates or at constant carbon capture rates. The resultant relationships can be further overlapped to create Combined Warming Index (CWI) Energy System Envelopes.

Our methodology provides a new structred approach to test the effects of methane leakage rate and carbon capture rate on complicated energy system models or integrated assessment models (IAMs). Even with this, there are some boundaries which should be respected. While energy system variables may can be interpolated within the limits of the Constant Methane Leakage Rate Trends, Constant Capture Rate Trends and Combined Warming Index (CWI) Energy System Envelopes, exprapolated results would not be relaible due to the non-linear relationships within energy system type models. There is a potential to further develop the methodology and apply it beyond our focus of blue hydrogen production from natural gas within the energy system. Other processes that involve natural gas or methane and apply CCS solutions to limit carbon doixide emissions would also be candidates for the application of this new technique. These include power generation from natural gas with a CCS component and boienergy with CCS.

## Ethics statements

Not applicable.

## Declaration of interests

The authors declare that they have no known competing financial interests or personal relationships that could have appeared to influence the work reported in this paper.

## Data Availability

Data will be made available on request.

## Appendix Supplementary materials

Image, application 1

Image, application 2

Image, application 3

Image, application 4

Image, application 5

Image, application 6

## Footnotes

1 Global Warming Potential (GWP) indicates the impact a gas will have on atmospheric warming over timescales when contrasted to carbon dioxide. CH4 GWP=25 and CO2 GWP=1 utilised in our estimations.

2 Detailed Life Cycle Assessment (LCA) is an important method to accurately estimate the total carbon intensity of blue hydrogen, covering all stages of the blue hydrogen supply chain and process.
