# Supplementary material for: Combined warming index energy system analysis framework for methane leakage rate and carbon capture rate uncertainty
Source: MethodsX. 2025 Jul 23;15:103526. doi: 10.1016/j.mex.2025.103526 (PMC12329510; doi:10.1016/j.mex.2025.103526)
Supplement: Supplementary file 1 [file mmc1.pptx]

## Slide 1
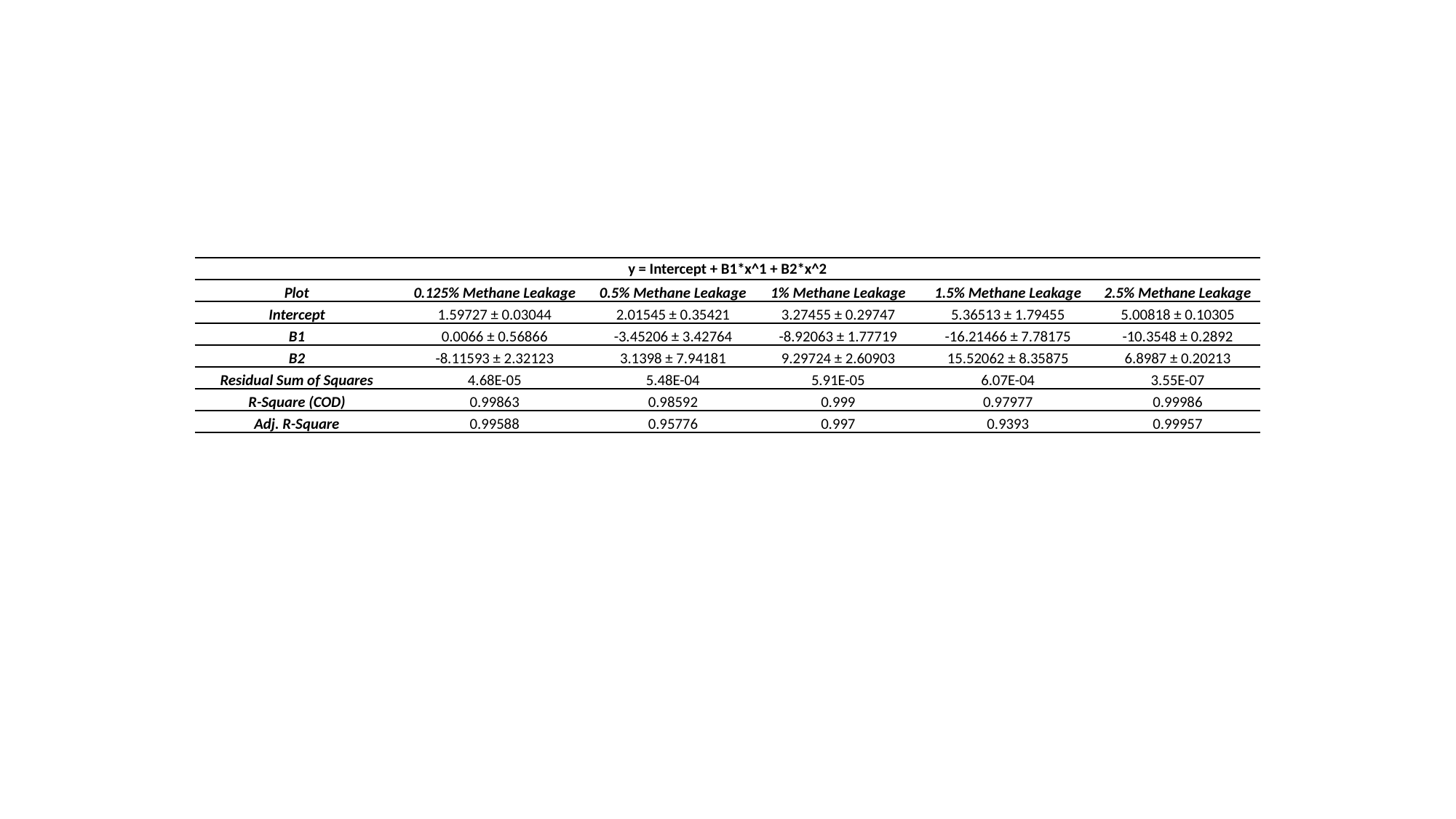

| y = Intercept + B1\*x^1 + B2\*x^2 | | | | | |
| --- | --- | --- | --- | --- | --- |
| Plot | 0.125% Methane Leakage | 0.5% Methane Leakage | 1% Methane Leakage | 1.5% Methane Leakage | 2.5% Methane Leakage |
| Intercept | 1.59727 ± 0.03044 | 2.01545 ± 0.35421 | 3.27455 ± 0.29747 | 5.36513 ± 1.79455 | 5.00818 ± 0.10305 |
| B1 | 0.0066 ± 0.56866 | -3.45206 ± 3.42764 | -8.92063 ± 1.77719 | -16.21466 ± 7.78175 | -10.3548 ± 0.2892 |
| B2 | -8.11593 ± 2.32123 | 3.1398 ± 7.94181 | 9.29724 ± 2.60903 | 15.52062 ± 8.35875 | 6.8987 ± 0.20213 |
| Residual Sum of Squares | 4.68E-05 | 5.48E-04 | 5.91E-05 | 6.07E-04 | 3.55E-07 |
| R-Square (COD) | 0.99863 | 0.98592 | 0.999 | 0.97977 | 0.99986 |
| Adj. R-Square | 0.99588 | 0.95776 | 0.997 | 0.9393 | 0.99957 |
