# Supplementary material for: Combined warming index energy system analysis framework for methane leakage rate and carbon capture rate uncertainty
Source: MethodsX. 2025 Jul 23;15:103526. doi: 10.1016/j.mex.2025.103526 (PMC12329510; doi:10.1016/j.mex.2025.103526)
Supplement: Supplementary file 2 [file mmc2.pptx]

## Slide 1
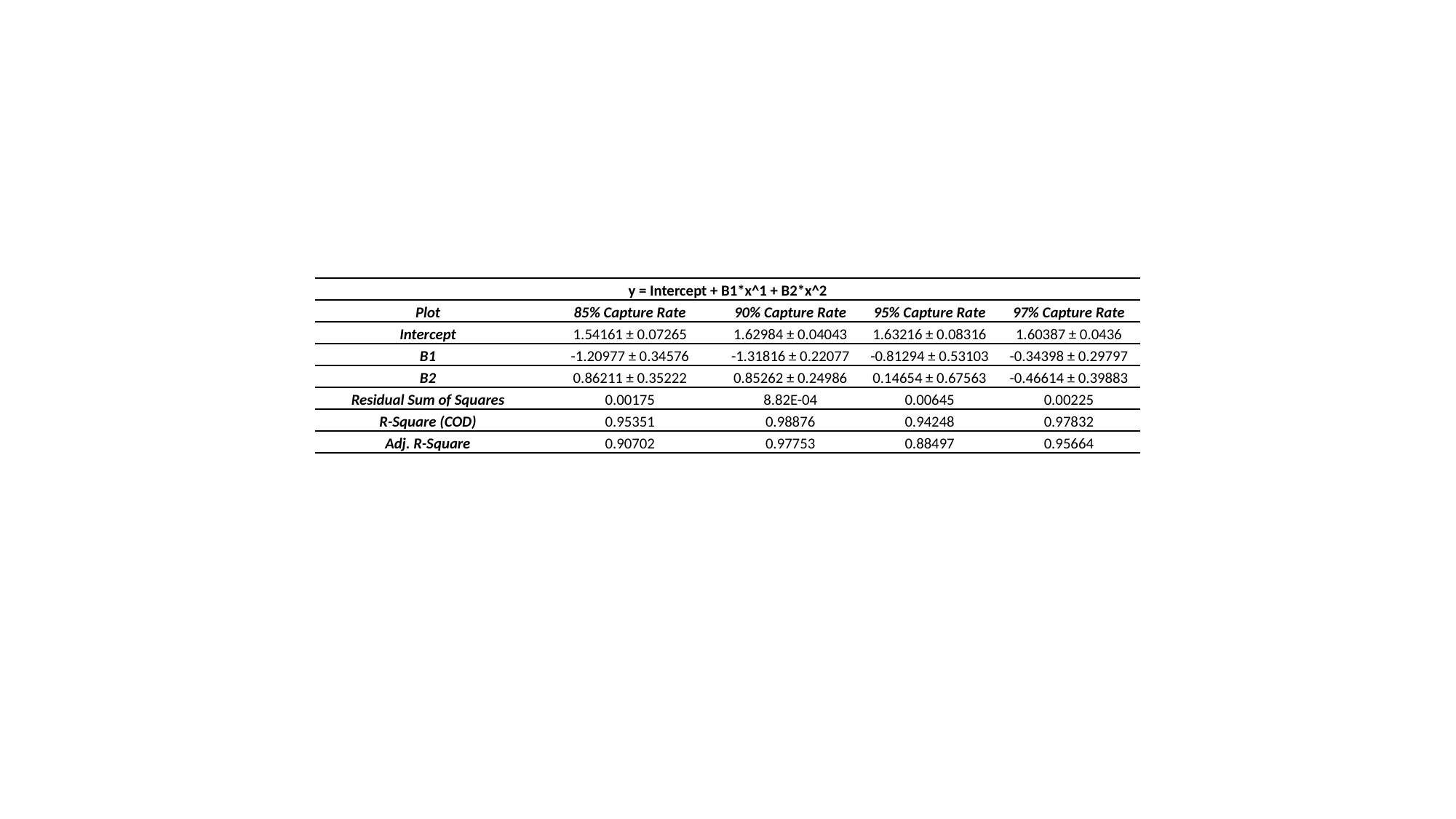

| y = Intercept + B1\*x^1 + B2\*x^2 | | | | |
| --- | --- | --- | --- | --- |
| Plot | 85% Capture Rate | 90% Capture Rate | 95% Capture Rate | 97% Capture Rate |
| Intercept | 1.54161 ± 0.07265 | 1.62984 ± 0.04043 | 1.63216 ± 0.08316 | 1.60387 ± 0.0436 |
| B1 | -1.20977 ± 0.34576 | -1.31816 ± 0.22077 | -0.81294 ± 0.53103 | -0.34398 ± 0.29797 |
| B2 | 0.86211 ± 0.35222 | 0.85262 ± 0.24986 | 0.14654 ± 0.67563 | -0.46614 ± 0.39883 |
| Residual Sum of Squares | 0.00175 | 8.82E-04 | 0.00645 | 0.00225 |
| R-Square (COD) | 0.95351 | 0.98876 | 0.94248 | 0.97832 |
| Adj. R-Square | 0.90702 | 0.97753 | 0.88497 | 0.95664 |
