# Supplementary material for: Combined warming index energy system analysis framework for methane leakage rate and carbon capture rate uncertainty
Source: MethodsX. 2025 Jul 23;15:103526. doi: 10.1016/j.mex.2025.103526 (PMC12329510; doi:10.1016/j.mex.2025.103526)
Supplement: Supplementary file 3 [file mmc3.pptx]

## Slide 1
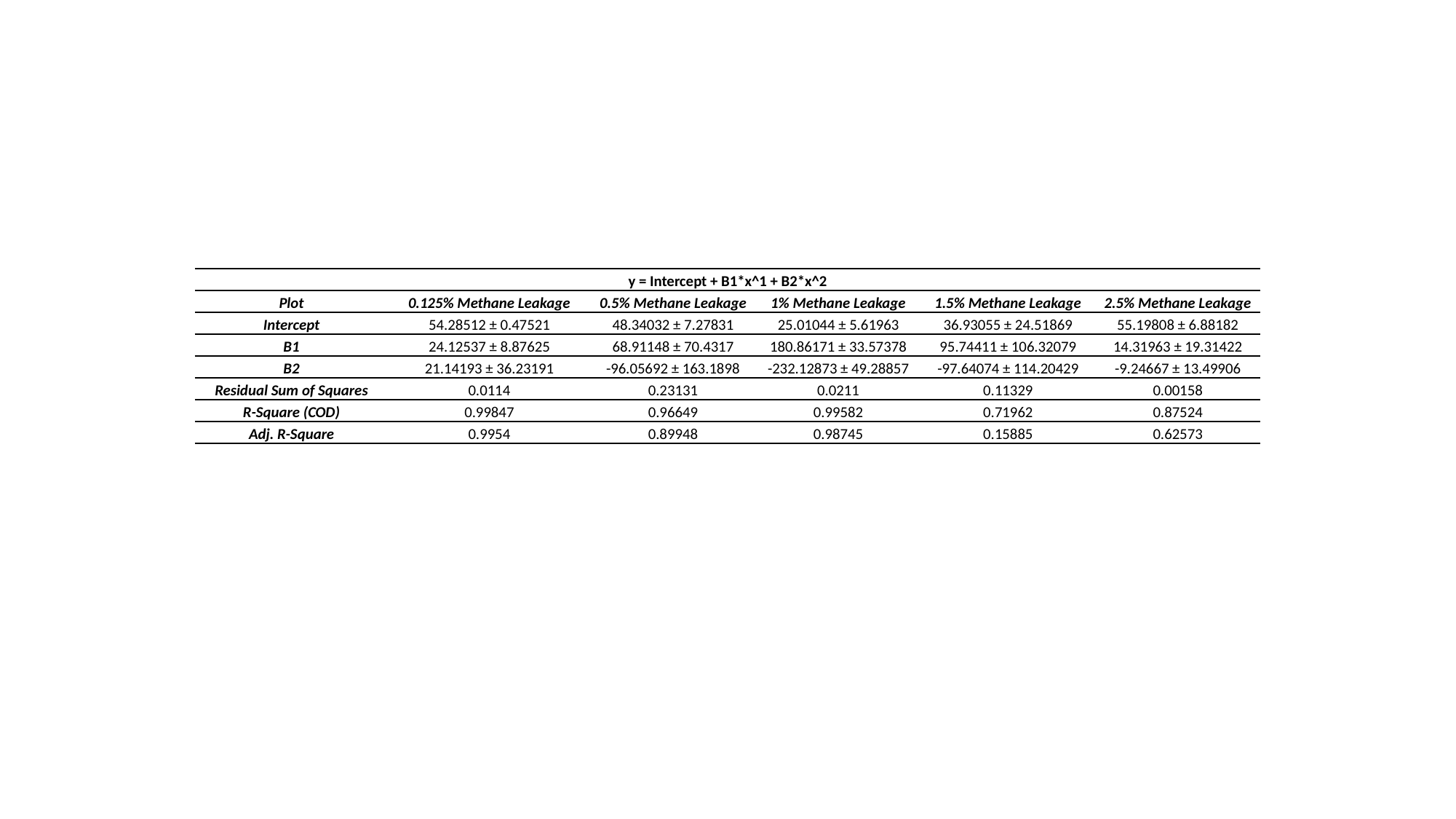

| y = Intercept + B1\*x^1 + B2\*x^2 | | | | | |
| --- | --- | --- | --- | --- | --- |
| Plot | 0.125% Methane Leakage | 0.5% Methane Leakage | 1% Methane Leakage | 1.5% Methane Leakage | 2.5% Methane Leakage |
| Intercept | 54.28512 ± 0.47521 | 48.34032 ± 7.27831 | 25.01044 ± 5.61963 | 36.93055 ± 24.51869 | 55.19808 ± 6.88182 |
| B1 | 24.12537 ± 8.87625 | 68.91148 ± 70.4317 | 180.86171 ± 33.57378 | 95.74411 ± 106.32079 | 14.31963 ± 19.31422 |
| B2 | 21.14193 ± 36.23191 | -96.05692 ± 163.1898 | -232.12873 ± 49.28857 | -97.64074 ± 114.20429 | -9.24667 ± 13.49906 |
| Residual Sum of Squares | 0.0114 | 0.23131 | 0.0211 | 0.11329 | 0.00158 |
| R-Square (COD) | 0.99847 | 0.96649 | 0.99582 | 0.71962 | 0.87524 |
| Adj. R-Square | 0.9954 | 0.89948 | 0.98745 | 0.15885 | 0.62573 |
