# Supplementary material for: Combined warming index energy system analysis framework for methane leakage rate and carbon capture rate uncertainty
Source: MethodsX. 2025 Jul 23;15:103526. doi: 10.1016/j.mex.2025.103526 (PMC12329510; doi:10.1016/j.mex.2025.103526)
Supplement: Supplementary file 4 [file mmc4.pptx]

## Slide 1
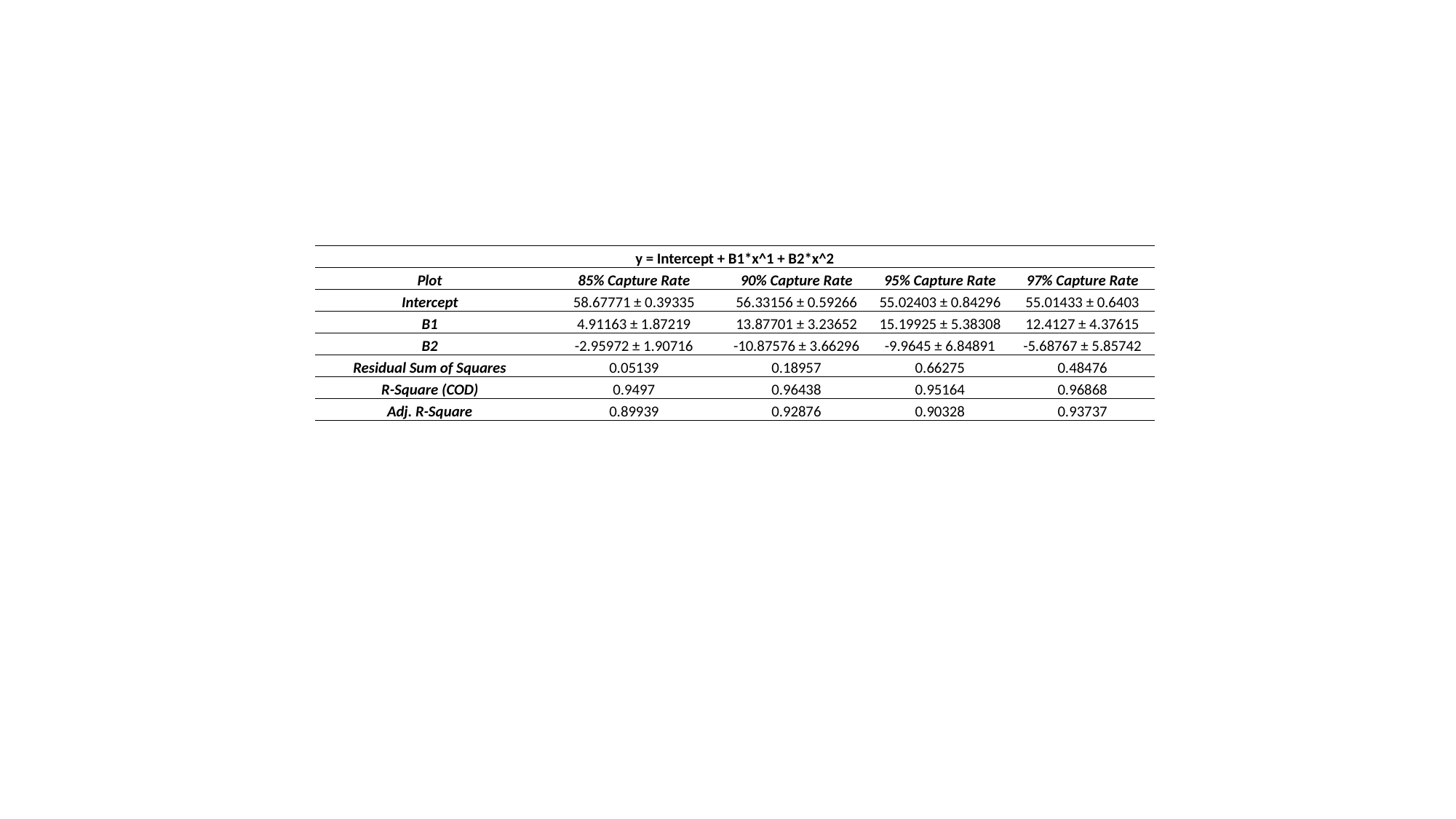

| y = Intercept + B1\*x^1 + B2\*x^2 | | | | |
| --- | --- | --- | --- | --- |
| Plot | 85% Capture Rate | 90% Capture Rate | 95% Capture Rate | 97% Capture Rate |
| Intercept | 58.67771 ± 0.39335 | 56.33156 ± 0.59266 | 55.02403 ± 0.84296 | 55.01433 ± 0.6403 |
| B1 | 4.91163 ± 1.87219 | 13.87701 ± 3.23652 | 15.19925 ± 5.38308 | 12.4127 ± 4.37615 |
| B2 | -2.95972 ± 1.90716 | -10.87576 ± 3.66296 | -9.9645 ± 6.84891 | -5.68767 ± 5.85742 |
| Residual Sum of Squares | 0.05139 | 0.18957 | 0.66275 | 0.48476 |
| R-Square (COD) | 0.9497 | 0.96438 | 0.95164 | 0.96868 |
| Adj. R-Square | 0.89939 | 0.92876 | 0.90328 | 0.93737 |
