# Supplementary material for: Combined warming index energy system analysis framework for methane leakage rate and carbon capture rate uncertainty
Source: MethodsX. 2025 Jul 23;15:103526. doi: 10.1016/j.mex.2025.103526 (PMC12329510; doi:10.1016/j.mex.2025.103526)
Supplement: Supplementary file 5 [file mmc5.pptx]

## Slide 1
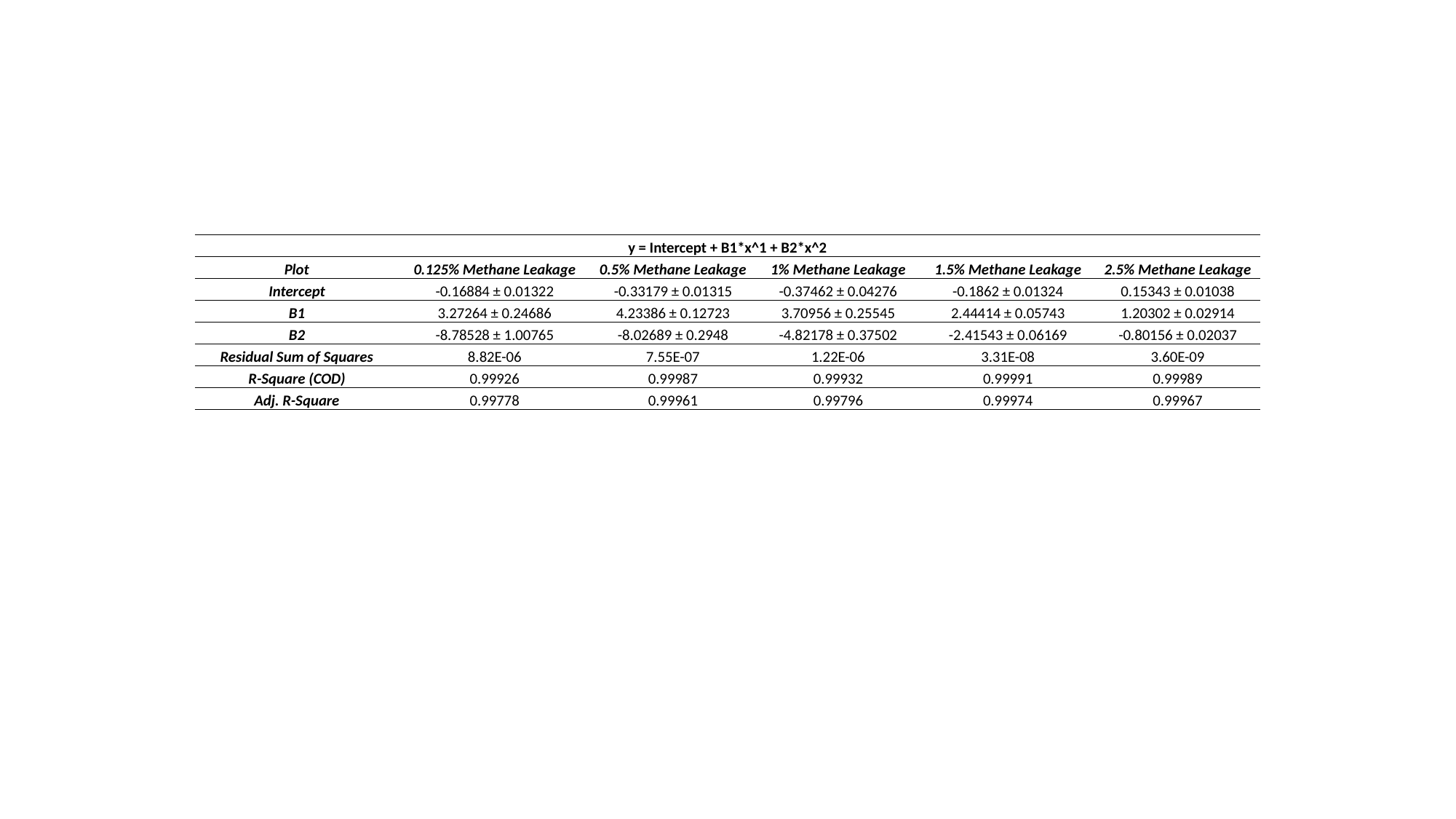

| y = Intercept + B1\*x^1 + B2\*x^2 | | | | | |
| --- | --- | --- | --- | --- | --- |
| Plot | 0.125% Methane Leakage | 0.5% Methane Leakage | 1% Methane Leakage | 1.5% Methane Leakage | 2.5% Methane Leakage |
| Intercept | -0.16884 ± 0.01322 | -0.33179 ± 0.01315 | -0.37462 ± 0.04276 | -0.1862 ± 0.01324 | 0.15343 ± 0.01038 |
| B1 | 3.27264 ± 0.24686 | 4.23386 ± 0.12723 | 3.70956 ± 0.25545 | 2.44414 ± 0.05743 | 1.20302 ± 0.02914 |
| B2 | -8.78528 ± 1.00765 | -8.02689 ± 0.2948 | -4.82178 ± 0.37502 | -2.41543 ± 0.06169 | -0.80156 ± 0.02037 |
| Residual Sum of Squares | 8.82E-06 | 7.55E-07 | 1.22E-06 | 3.31E-08 | 3.60E-09 |
| R-Square (COD) | 0.99926 | 0.99987 | 0.99932 | 0.99991 | 0.99989 |
| Adj. R-Square | 0.99778 | 0.99961 | 0.99796 | 0.99974 | 0.99967 |
