# Supplementary material for: Combined warming index energy system analysis framework for methane leakage rate and carbon capture rate uncertainty
Source: MethodsX. 2025 Jul 23;15:103526. doi: 10.1016/j.mex.2025.103526 (PMC12329510; doi:10.1016/j.mex.2025.103526)
Supplement: Supplementary file 6 [file mmc6.pptx]

## Slide 1
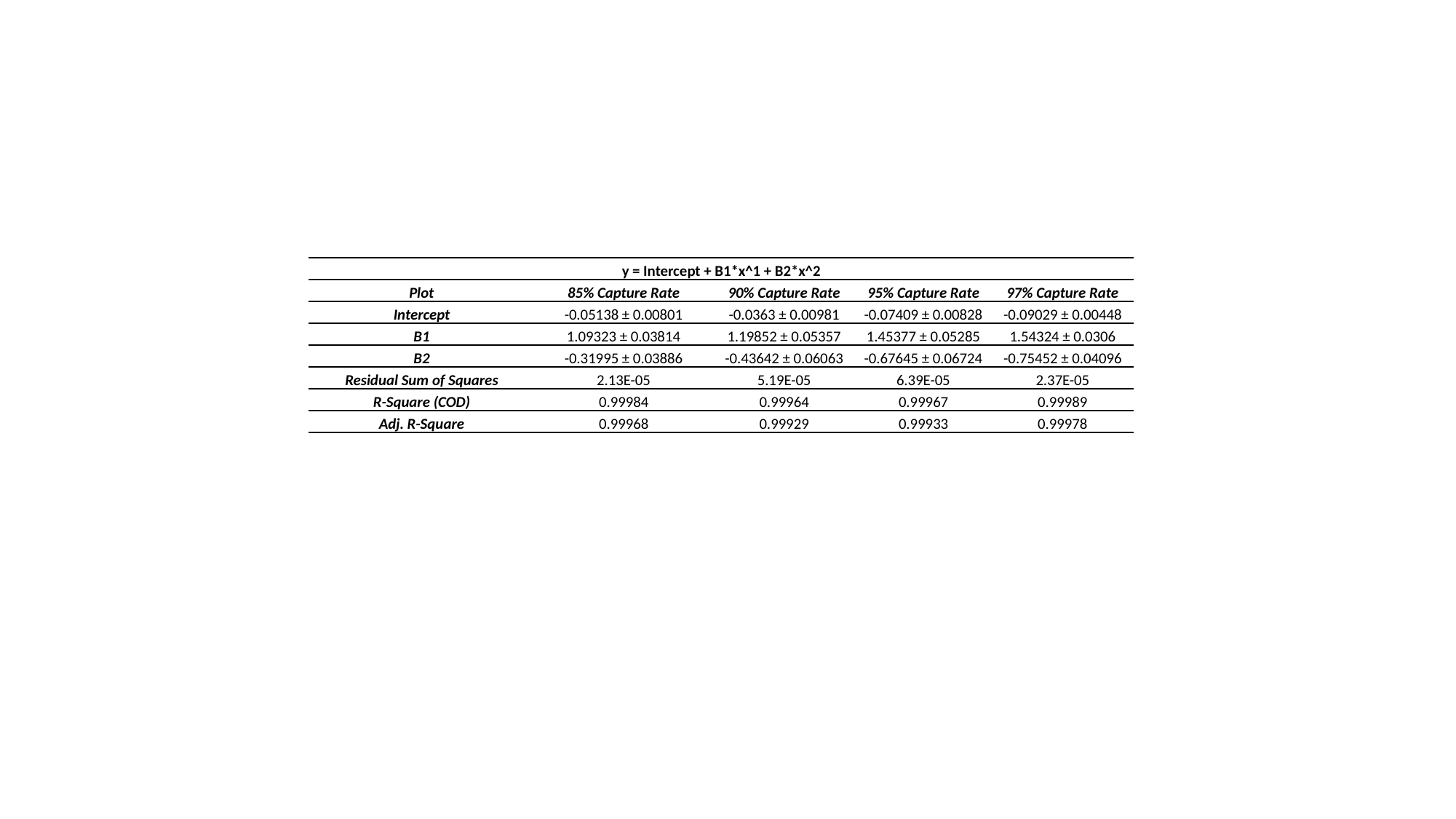

| y = Intercept + B1\*x^1 + B2\*x^2 | | | | |
| --- | --- | --- | --- | --- |
| Plot | 85% Capture Rate | 90% Capture Rate | 95% Capture Rate | 97% Capture Rate |
| Intercept | -0.05138 ± 0.00801 | -0.0363 ± 0.00981 | -0.07409 ± 0.00828 | -0.09029 ± 0.00448 |
| B1 | 1.09323 ± 0.03814 | 1.19852 ± 0.05357 | 1.45377 ± 0.05285 | 1.54324 ± 0.0306 |
| B2 | -0.31995 ± 0.03886 | -0.43642 ± 0.06063 | -0.67645 ± 0.06724 | -0.75452 ± 0.04096 |
| Residual Sum of Squares | 2.13E-05 | 5.19E-05 | 6.39E-05 | 2.37E-05 |
| R-Square (COD) | 0.99984 | 0.99964 | 0.99967 | 0.99989 |
| Adj. R-Square | 0.99968 | 0.99929 | 0.99933 | 0.99978 |
